# ‘Young People Just Resolve It in Their Own Group’: Young People’s Perspectives on Responses to Non-Consensual Intimate Image Distribution

**DOI:** 10.1177/14732254211030570

**Published:** 2021-07-09

**Authors:** Alexa Dodge, Emily Lockhart

**Keywords:** criminal law, education, non-consensual intimate image distribution, non-consensual pornography, restorative justice, revenge porn, youth justice

## Abstract

While responses to non-consensual intimate image distribution (NCIID) often highlight criminal law remedies, little is known about how young people are choosing to respond to this act and whether they perceive legal intervention as a useful tool. Drawing from interviews with 10 teenagers and survey responses from 81 adult supporters, we provide insight into how young people perceive the supports available to them for responding to NCIID. We find young people may avoid seeking support from both the criminal justice system and adults in general due to fears of adult overreaction, victim blaming and shaming, and self/peer criminalization.

## Introduction

The act of distributing nude or sexually explicit images without consent – often colloquially referred to as ‘revenge porn’ or ‘non-consensual pornography’ – has become an issue of popular concern and the product of frequent news headlines internationally (Buiten, 2020; Powell and Henry, 2017). Over the past decade, this act has increasingly attracted widespread social concern as a result of a number of reported cases in which victims experienced – sometimes severe – psychological harms, reputational harms (based on sex-negative cultural beliefs about sexual exposure), economic harms and/or harms related to their exposed image being used as fodder for bullying and harassment (Powell and Henry, 2017; Dodge, 2021b). Much of the public outcry, activism and government response to non-consensual intimate image distribution (NCIID)^1^ has centred on calls to criminalize this act (Citron and Franks, 2014; Hill, 2015; Kitchen, 2015). These calls for a criminal justice response have resulted in the creation of specific criminal offences for NCIID in jurisdictions such as Canada, parts of the United States, the United Kingdom, the Philippines, Israel, Australia and Japan (Crofts and Kirchengast, 2019; Powell and Henry, 2017).

While criminalization will likely help to denounce and deter this act to some extent and offer a portion of victims the recourse they seek (Citron and Franks, 2014; Hill, 2015; Kitchen, 2015), a cohort of NCIID scholars have argued that criminal law is limited in its ability to support the needs of many victims and may be particularly ineffective in meeting the needs of young people (Powell and Henry, 2017; Shariff and DeMartini, 2015; Dodge and Spencer, 2018). While calls to criminalize this act often spotlight cases involving young people – specifically those cases with the most tragic outcomes^2^ – little research has been done to assess how young people are choosing to respond to this act in practice and whether they perceive legal intervention as a useful tool. Based on interviews with 10 teenagers and open-ended survey responses from 81 adults who work closely with teens, in this article we provide insight into how young people perceive the supports available to them for responding to NCIID. We find that young people may avoid seeking support from both the criminal justice system and adults in general due to: feeling that most cases can be dealt with by young people at the peer level and that adults will ‘overreact’; concerns that adults will engage in victim blaming and shaming; and concerns that victims or perpetrators will be criminalized.

This article is composed of four sections. We begin with an overview of research by feminist and restorative justice scholars on the limits of criminal responses to NCIID among young people. In the second section, we provide the methodology for our interviews and survey data. In the third section, we discuss our findings regarding young people’s perspectives on responses to NCIID. Based on our research findings, in the final section, we argue that it is necessary to make non-criminal supports widely available to young people and to ensure that supports are provided in a non-judgemental manner that challenges common victim blaming and shaming narratives. Young people have been largely excluded from the social processes through which knowledge about them is produced (Best, 2007); we argue that understanding young people’s perspectives on criminal responses to NCIID will allow for the development of resources that they are more likely to engage with and that are more responsive to their stated needs.

## Criminal and Alternative Responses to NCIID Among Young People

Emerging research finds that criminal justice responses may fail to address both the most pressing needs of young NCIID victims and the root causes of this act among young people (e.g. lack of knowledge regarding sexual consent, sexual rumour spreading practices, sexist bonding practices, and the normalization of sexist, sex negative and gender-norm enforcing bullying; Bailey, 2014; Coburn et al., 2015; Crofts and Lievens, 2018; Shariff and DeMartini, 2015; Dodge, 2021a). As Henry et al. (2017) argue, criminal responses may fail to meet the needs of NCIID victims of all ages as they are aimed at establishing guilt and punishing perpetrators on behalf of the state rather than on providing these victims with ‘the remedies that they need, such as advice and counseling, and most importantly, takedown of non-consensual imagery’ (p. 8). Providing initial evidence of young people’s perspectives on criminal responses, research with Canadian police officers who respond to acts of NCIID found that the teenage victims they interact with are generally not interested in engaging a criminal justice response; rather, they are looking for alternative supports to have their intimate image expediently deleted and to address the discriminatory beliefs among their peers that can fuel widespread victim blaming and shaming (Dodge and Spencer, 2018). As criminal responses are focused on investigating and punishing particular individuals, rather than transforming discriminatory student cultures and supporting victims, they may be ill-suited to respond to the needs of many victims and the complex interpersonal dynamics and various sources of bullying/harm that arise in cases of NCIID among young people.

The popular focus on criminal law responses as the solution to NCIID can act to sideline discussions regarding the potential for non-criminal responses to express societal denunciation of this behaviour and to address the varying needs of victims, perpetrators and communities in the aftermath of non-consensual distribution (Hamilton, 2018; Powell and Henry, 2017; Shariff and DeMartini, 2015; Dodge, 2021a). Recognizing the shortcomings of criminal justice–focused responses, many NCIID scholars have taken up feminist and restorative justice theories to argue for the effectiveness of responses based on restorative or transformative justice principles, victim-centred supports and sex-positive consent education (that actively undermines victim blaming and shaming beliefs; Albury et al., 2016; Crofts and Lievens, 2018; Hamilton, 2018; Henry et al., 2017; Shariff and DeMartini, 2015; Dodge, 2021b). These arguments for alterative responses to NCIID align with anti-carceral feminist and restorative justice scholarship in the area of sexual violence more broadly; this scholarship posits that the punitive, perpetrator-focused and often lengthy processes offered by the typical criminal justice response are at odds with the core wants and needs of many victims of sexual violence (Bernstein, 2012; Bumiller, 2008; Taylor, 2018).

There are initial indications in the scant research that young people may be interested in avoiding criminal responses to NCIID and are seeking alternative responses (Dodge and Spencer, 2018); however, there is no research to our knowledge that engages the voices of young people themselves in the question of criminal responses to this issue. In this article, we draw on the voices of young people and those adults who work closely with young people around image-sharing practices to better understand if, how and under what circumstances teenagers might mobilize criminal law in cases of NCIID and why they may prefer alternatives. We use feminist scholarship regarding criminal, educational and alterative responses to sexual violence generally and NCIID specifically to question the adequacy of criminal responses to NCIID among young people; in addition, we use restorative justice scholarship to imagine how responses to NCIID might be better addressed through approaches such as restorative and transformative justice, education-based cultural change and broader social justice movements (Hamilton, 2018). As Taylor (2018) explains, ‘while it is a fact that some survivors of sexual violence, such as those involved in victims’ rights movements, do seek retribution for the harms done to them’, many survivors are interested in accessing supports outside of the criminal justice system that are better able to meet their needs in terms of ‘recognition and validation of their stories, respect, dignity, voice, agency, an apology or accountability on the part of the person who harmed them, to feel safe again, and for what occurred not to happen to someone else’ (p. 13). As has been the case in regard to sexual violence scholarship more broadly, restorative justice scholarship and the scholarship of feminists who are critical of the criminal justice system are valuable for exploring the limits of criminal responses to NCIID among young people and for envisioning alternative responses.

## Method

### Interviews with young people

The data used in this article were collected by one of the authors in collaboration with youth organizations in the province of Nova Scotia, Canada. This geographical region was selected due to Nova Scotia’s central position in the fight to implement a federal NCIID law^3^ in Canada after the death of Rehtaeh Parsons and given that it was one of the first provinces to prosecute young people under Canada’s NCIID law (Bresge and Tutton, 2016).

In 2017, interviews were conducted with 10 young people between the ages of 14 and 17 living in Nova Scotia. Interview participants were recruited in collaboration with community organizations serving young people and through posters advertising the research that were circulated with the help of community youth organizations, Facebook, Instagram and Twitter. Participation was completely voluntary and no incentives were offered to those who were interviewed. In terms of demographic characteristics, of the 10 interviewees, 2 identified as male and 8 as female; 1 identified as queer and 9 as straight; 2 identified as Indigenous, 1 as Black, 1 as Multiracial and 6 as White. The interviews were semi-structured and included questions regarding young people’s perceptions of the act of NCIID, the efficacy of criminal law responses and the responses to incidents of NCIID that occur among their peers. All interviews were audio-recorded and transcribed in full. Interviewees were assigned pseudonyms to maintain their confidentiality. In terms of data analysis, open coding (Charmaz, 2006) was conducted using QSR NVivo qualitative research software to organize findings based on emerging themes (Leech and Onwuegbuzie, 2011). This article focuses on those themes related to perspectives on criminal and alternative responses to NCIID. Participants were asked to share their reactions to various scenarios of NCIID and to discuss how they and their peers tend to understand and respond to acts of NCIID at their schools. However, young people were not asked whether they had personally experienced being a victim of NCIID; therefore, we cannot ascertain whether participants had themselves experienced NCIID. We recognize this as a limitation of this study. We also recognize that the relatively small number of interviewees that were able to be recruited is a limitation of the study; however, additional data to support interview findings were gleaned from the survey responses described below. The interviews with 10 young people provide a deep and detailed engagement with a cohort of young people rather than broad and generalizable findings.

### Open-ended survey of young people’s supporters

The interviews described above provided detailed accounts of how these 10 young people understand the efficacy of various responses to NCIID; however, the number of young people that responded to calls for participation was relatively small and none of the interviewees disclosed having been personally victimized by this act. As such, a survey was conducted with 81 adults who work closely with young people to further understand how young people, and particularly those who have been victims of NCIID, are understanding and accessing supports in the aftermath of this act. The 81 survey respondents, or ‘supporters’, were made up of a range of service providers that work closely with young people across Nova Scotia, including teachers, youth workers, community youth programmers, restorative justice practitioners, coaches and school resource officers. The survey was distributed via email, LinkedIn, Facebook and Twitter. Respondents included 60 women, 18 men and 3 individuals who identified as gender non-conforming. Among these respondents, 66 indicated that they identified as straight, 3 as gay/lesbian and 12 as bisexual, pansexual or queer. In terms of racial diversity, 59 of the respondents self-identified as White, 3 as First Nations, 1 as Metis, 1 as African Canadian, 1 as Latino, 2 as multiracial and the others chose not to indicate their race or ethnicity. The survey asked nine questions related to intimate image-sharing practices among young people, asking respondents to provide insight based on their particular experiences as people who work closely with young people, including working with teens around the practice of image sharing. In this article, we focus on open-ended responses to the questions ‘in your experience, what do teens do when intimate images (nudes) are shared without permission/consent?’ and ‘how do you believe young people would want this act to be responded to?’ Responses to these open-ended survey questions provided additional data that supplemented our interview findings. The survey responses should also be understood as providing deep, qualitative engagement with the perspectives of respondents rather than broadly generalizable data.

## Young People’s Perspectives on Responding to NCIID

Our interview and survey findings highlight young people’s views of criminal law (and the adult world more generally) as a helping or non-helping site in incidences of NCIID. Teenage interviewees understood most acts of NCIID as something they were capable of handling at the peer level and as only in need of the intervention of an authority figure in certain – more extreme – situations. In response to questions about when they would engage the law, participants generally said that, in the majority of situations, they would not consult an adult at all, let alone seek to engage the criminal justice system. As one teenage interviewee explains,

[In most instances of NCIID] teens are doing their own things and kinda resolving things themselves and the only time it becomes an issue is when it’s something like really bad where someone is really getting harassed. [. . .] it’s not like you’re like, ‘ahhh so and so just sent my nude, I’m gonna go tell my mom’, ummm no. Students and young people just resolve it in their own group. (Audrey, age 16)

As Audrey’s comments evince, in contrast to popular framings of NCIID as *necessarily* causing extreme harm and requiring criminal justice interventions, our interviewees expressed that young people understand this act as a somewhat commonplace issue that they are capable of tackling on their own in the majority of instances.

Adults who work closely with young people likewise expressed that, from their perspective, young people are regularly dealing with NCIID individually or within their peer groups. Adult supporters were asked the open-ended question: ‘In your experience, what do teens do when intimate images (nudes) are shared without permission/consent?’ The majority of respondents explained that they believe young people ‘seek peer support’ (P 4) and ‘talk about it among themselves but usually don’t notify adults’ (P 64). Respondents expressed that young people only report to adults of any kind (let alone criminal justice personnel) in cases that are perceived as the most severe or as having gotten out of hand despite peer-level attempts to address the issue. While part of young people’s hesitancy to report can be explained by their expressed feeling that they are often capable of addressing it at the peer level, our research also found more complex reasons that young people may avoid reporting even in those cases where they feel more support is needed.

### Why young people avoid the law and other formal supports

Adult supporters provided a rather simplistic explanation for why they believe young people wish to avoid engaging adult resources. As one survey respondent puts it, ‘usually they keep it to themselves, or friends but usually don’t include adults – teachers or parents – [because] they don’t want to be seen as a “rat” or someone who can’t handle a “joke”’ (P 6). In contrast, the young interviewees describe a more complex picture of why they and their peers might be hesitant to report. In addition to seeing this act as capable of being dealt with at a peer level in many instances, young people explained that even in more severe situations they and their peers might avoid reporting due to concerns that adults would overreact, use criminal law in an unhelpful manner and/or blame and shame victims.

Young people’s concerns regarding victim blaming and shaming were top of mind for several interviewees. They explained that, as a young person, the possibility of judgemental adult responses is often understood as a worse fate than having other teens see one’s nude image. This is emphasized by one participant who asserts that, even if she was being bullied every day as a result of having an intimate image non-consensually distributed, she believes she would not report the act to an adult as she would not want her mother to be upset that she consensually shared a nude photograph at the outset (Audrey, age 16). Another participant felt that most young people would avoid telling their parents and explained,

some people have good relationships with their parents, but I mean I wouldn’t personally tell my mom about that first. But like I feel like she’d find out either way or like I’d tell her eventually if it got real bad. (Avery, age 17)

Audrey and Avery’s concerns regarding parental victim blaming and shaming may be well founded, as criminologists and education scholars have found that a lack of acceptance of young people’s sexuality and digital sexual practices often result in their acts of consensual image sharing being treated as abnormal, immoral or even criminal (Albury et al., 2016; Karaian and Van Meyl, 2015; Wodda and Panfil, 2018; Dodge, 2021b). In fact, both youth and adult victims regularly report that their biggest fear in the aftermath of image exposure is that their parents will find out about their consensual image distribution and be disappointed, angry with them or ashamed of them (McGlynn et al., 2017; Dodge, 2021b). Sex-positive feminist analysis of education campaigns directed at young people finds that these campaigns often communicate the belief that consensual youth sexting is dumb, dirty or dangerous and, thereby, reaffirms rather than questions victim blaming and shaming scripts in response to NCIID among young people (Albury et al., 2016; Angelides, 2013; Naezer and van Oosterhout, 2020). A recent example of the ways education campaigns can normalize victim blaming and shaming behaviours can be seen in education materials from the Canadian Centre for Child Protection that warns young people who consensually shared images that were later shared without consent to

be aware that your parents/safe adult are likely to feel a wide range of emotions hearing that you have created and shared a sexual picture/video of yourself with peers. This may include disappointment, anger or hurt . . . It is normal for your family to be feeling these things and more when they receive this type of news.^4^

Such warnings make it perhaps unsurprising that interviewees perceive reporting to adults as an unappealing option. Our findings in this regard offer additional support to sex-positive feminist arguments that putting the onus on potential victims to abstain from consensual intimate image sharing, rather than using rights-based education to teach potential perpetrators about consent and bystanders about the detrimental impacts of victim blaming and shaming, can result in reaffirming rather than undermining victim blaming and shaming beliefs (Naezer and van Oosterhout, 2020; Setty, 2018).

Related to fears of victim blaming and shaming, many interviewees also expressed that victims might not report to adults due to fears of being criminalized themselves. Despite the fact that a young person has never been convicted for *consensual* intimate image sharing (i.e. sexting) in Canada, and legal scholars widely believe they likely never will be/should never be (Karaian and Brady, 2020), some young people reported receiving information during school assemblies that consensually sending your own nudes was considered child pornography and could result in criminalization. Avery explained this experience at her school:

There was a social worker and a police officer there that were talking to us about it and like saying that sending nudes, that’s like, if you send your own nude that’s still distributing child pornography so . . . I’m pretty sure half the people there sent nudes themselves and no one was trying to say that cause they were probably like oh there’s a police officer right there and they’re gonna arrest me in the middle of the gym. (Avery, age 17)

Due to victim responsibilizing narratives provided by police officers and others through education resources for young people (even in countries where consensual image sharing has not been criminalized in practice; Angelides, 2013), the fear of being criminalized themselves provides yet another reason that young people may avoid engaging adult supports and are especially concerned with involving criminal justice personnel in any way. It is striking to imagine the moment in which teens who have consensually shared images hear the, likely frightening, message that they have unknowingly committed a crime (and the highly stigmatizing crime of child pornography at that) (Karaian and Brady, 2020; Shariff and DeMartini, 2015). While such warnings may be well-intentioned (though ill-advised) attempts to scare young people out of consensual image sharing so as to lower the risk of NCIID victimization, it is clear that it has the effect of communicating to young people that, if they shared the image consensually at the outset, they should deal with NCIID on their own to avoid self-incrimination. Given the responsibilizing messages presented in anti-sexting campaigns (Angelides, 2013; Karaian, 2014), representations of sexting-related criminal charges on television (Lockhart, 2018) as well as the fact that minors have been charged for sending their own nudes in some jurisdictions in the United States (Graw-Leary, 2010), this finding (while concerning) is not surprising. It is clear that much work is needed to effectively communicate the complex criminal laws regarding consensual sexting versus NCIID to young people and to send the clear message that victims will be supported rather than blamed, shamed or criminalized.

Many teenage interviewees also expressed that they believe young people are unlikely to formally invoke the law, or report to an adult that may engage the law, due to a desire to avoid criminalizing their peers. These interviewees stated that criminalizing peers is seen as an overreaction that would not help to address the needs of victims. This finding is in keeping with research on police perspectives that revealed that police believe teenage victims often do not want to criminalize their peers or engage in lengthy criminal justice processes, rather they want support to have pictures deleted expediently and related, sometimes widespread, peer bullying addressed (Dodge and Spencer, 2018). One interviewee, Aaron, expressed that he believes his peers avoid seeking the supports of the criminal justice system even in serious cases due to this hesitancy to criminalize peers. He shared a story about one of his female friends who was non-consensually photographed having sex and the photo was shared without her consent:

It’s not because she doesn’t trust the legal system [that she didn’t report], it’s because she doesn’t want people to get in trouble for it. She’s thinking that they’re young, they’ve done it but they shouldn’t do it again. She’s trusting them, I’d say too much in my opinion. (Aaron, age 16)

While Aaron believes more formal intervention is needed here to ensure that the perpetrator does not commit such acts in the future, he describes the victim herself in this case as wanting to avoid engaging with the law as she does not want the perpetrator to get in trouble. Aaron’s comments highlight the fact that young people may want/need the support of adults in some cases of NCIID, but may choose not to seek support even when they are in over their heads due to fears of the perpetrator being criminalized or otherwise harshly punished rather than educated on their behaviour being wrong. From this perspective, young people might be more willing to seek support if they knew they would be able to gain resources without losing control over how their case is dealt with. For these young people, the ‘adult world’s’ framing of NCIID as always an extreme issue requiring criminal intervention may result in young people avoiding seeking adult support due to fears that adults will force them into a criminal justice response or otherwise (from their perspective) overreact.

### Challenges to responding at the peer level

Due to the above concerns, our teenage interviewees explained that they would most likely deal with these kinds of situations themselves or within their peer groups. While some young people may have the knowledge and skills to support their peers, our interviewee responses suggest that tactics taken by young people can range from seemingly helpful emotional support for victims and attempts to educate perpetrators on consent to unhelpful behaviours of fighting and name-calling. Seventeen-year-old Feara viewed herself as an advocate and a problem solver, taking great pride in these skills. Feara explained that she would help friends if their images were shared without consent by giving them advice about how to get them back or confronting the person who shared them:

if it’s a close friend of mine, I’d get to the bottom of it and tell [the perpetrator] they shouldn’t be doing this or this person only gives consent to show you not everybody else so you should respect their trust. You should respect what they asked for. (Feara, age 17)

Many teenage participants explained that they would resolve the issue on their own by challenging the sharer directly. Adult supporters echoed this finding expressing that, in their experience, young people often ‘challenge the sharer directly’ (P 27) and ‘confide in friends and use social media to confront the people who did it’ (P 66). A conversation with 17-year-old Avery revealed that doing this in a manner that does not escalate tensions can sometimes be challenging for young people:

Interviewer: Do you think youth are kinda handling these things [instances of NCIID] on their own?

Avery: Oh ya that happens all the time. It will just, there will be sides taken and then everyone will just fight until someone either leaves the school or it just dies down. Like it eventually dies down but then someone always brings it back up again like 2 months later.

Interviewer: Right. But there’s no like, there’s no plan amongst youth about like how we’re gonna deal with this like can we sit down and mediate this situation on our own?

Avery: There’s no planning. There’s just fighting and name-calling.

Avery’s example is an interesting contrast to that described by Feara; while Feara understood her approach as a constructive attempt to educate the perpetrator on consent, Avery’s experience suggests that it is important for young people to have the tools to know what behaviour is useful and what could escalate the issue. Many adult supporters expressed their belief that these peer-level attempts may raise tensions and fail to address the issue constructively. As one survey respondent put it, young people ‘complain to their friends and plot revenge, but don’t consult anyone who can assist them in taking positive action that will actually help them’ (P 2). While it is important to account for the fact that young people might feel more supported by a friend like Feara than by a potentially victim blaming adult, it is also clear here that young people do not always have the tools to respond to NCIID adequately. However, as described above, young people may be unlikely to seek the support of adults due to a framing of this act at the adult level as something shameful (and potentially criminal) on the part of victims and as always something requiring a criminal or severe intervention in response to perpetrators.

Recognizing that young people’s attempts to resolve these situations can result in intergroup conflict, adult respondents express wanting to find alternative ways to support young people. While expressing that criminal law is of limited use in these situations and should be used only in the most extreme cases, supporters saw young people as having a limited ability to effectively respond to this act on their own and, therefore, thought that alternative responses should be made available. Adult supporters were asked to share what they believe justice looks like for young victims of NCIID. Generally, respondents believe that responses should be ‘quick and confidential’ (P 1); provide the victim with ‘ample connections to professionals who specialize in alleviating the effects of these circumstances’ (P 2); be ‘individualized’ (P 5) and ‘self-determined by the victim/survivor’ (P 4); and focused on restorative or transformative approaches in most cases. Some respondents thought that certain cases might deserve a criminal justice approach if the act was particularly ‘malicious’ (P 21), but generally respondents advocated for non-criminal responses including ‘education and empathy work for the perpetrator’ (P 15) and a process that supports victim healing rather than re-traumatization. For some respondents, these victim-centred approaches included perpetrators being ‘held accountable in a way that is meaningful to the victim and the surrounding community’ (P 22) and ensuring that victim supports are available in ‘school, their community, and family to ensure there is no shame’ (P 1). While it seems likely that such alternative responses would be more appealing to young people than criminal justice options, much groundwork is needed to create a cultural context in which young people feel that they can seek adult supports without being exposed to victim blaming and shaming or insighting a response that they perceive as an unhelpful overreaction.

## Discussion and Conclusion: Non-Criminal and Non-Judgemental Responses

The above findings align with the scant existing evidence that young people may not view formal criminal law responses as a useful tool in responding to NCIID (Dodge and Spencer, 2018).^5^ Our interviewees suggest that young people may perceive this act as something they can deal with at the peer level and, when more severe cases arise, they may still avoid seeking help from an adult (let alone criminal justice officials) due to a perceived lack of non-judgemental and helpful supports. Our interviewees provide evidence that victim blaming and shaming narratives along with unclear education regarding the potential criminalization of victims may result in young people avoiding seeking adult supports. In addition, interviewees express that young people may avoid reporting to adults due to a reluctance to criminalize their peers for an act that, in my cases, they view as not requiring such an ‘overreaction’ from the adult world. Together, these findings suggest that it is necessary to continue developing non-criminal alternative responses to NCIID, as argued by several scholars in this area (Hamilton, 2018; Shariff and DeMartini, 2015), and to ensure that supports and education are non-judgemental and actively undermine victim blaming and shaming narratives, as suggested by sex-positive feminist scholars.

Feminist scholars, and especially sex-positive feminist scholars, offer valuable insight for addressing the fears of adult victim blaming and shaming expressed by our teenage interviewees. These scholars argue that, in order to avoid reaffirming the sex-negative beliefs that fuel victim blaming and shaming, education on and responses to NCIID *must* accept consensual image sharing as a legitimate sexual act that, like all sexual acts, comes with both potential pleasures and potential risks (Albury et al., 2016). In the context of young people, the ability to provide non-judgemental supports to victims of NCIID hinges on the ability to accept young people as sexual beings that should not be blamed or shamed for their involvement in consensual image sharing but, rather, should be clearly taught that they have the right to bodily autonomy and will be supported without judgement when others do not respect that right (Bivens and Fairbairn, 2015; Setty, 2018). When consensual image sharing is framed as a purely risky and inappropriate behaviour by adults (whether through risk-based educational approaches or warnings of potential criminal responses against victims), this ‘sex-as-danger truism’ (Khan, 2017, 351) leaves little room for young people to understand sexual consent and their right to bodily autonomy (Dodge, 2021b). Responding to this issue, several sex-positive feminist scholars have discussed the necessity of creating educational responses and supports for young people that recognize them as sexual beings and stress that it is when intimate image sharing, or any other sexual activity for that matter, is done without consent that it becomes problematic (Hasinoff, 2015; Naezer and van Oosterhout, 2020; Wodda and Panfil, 2018). As Naezer and van Oosterhout (2020) find, the taboo against consensual sexting results in young people, parents and teachers judging young people for participating in sexting which can actually make ‘it easier to share images without the maker’s consent and to blame the victim’ (p. 9). Sex-positive approaches are helpful in challenging social norms that lead to victim blaming and shaming and could, therefore, begin to create a culture in which young people are both more likely to feel comfortable seeking adult supports and more likely to advocate for, rather than shame and blame, their peers when dealing with these acts at a peer level. However, the first step in creating a sex-positive narrative around this issue is a difficult one, as it will require that adults (teachers, parents, counsellors, social workers, alternative justice providers and criminal justice personnel) interrogate their own sex-negative beliefs in order to make good on the promise of providing non-judgemental supports to teenage victims. In addition, the law must be changed in those jurisdictions where victims who consensually share intimate images can be criminalized as child pornographers (Karaian and Brady, 2020).

Both the young people and their supporters in our study shared that they believe young people are often dealing with instances of NCIID at a peer level. Thus, as we work towards creating cultures in which young people feel more comfortable seeking support from adults, it is also important to acknowledge that many teenagers are currently not seeking adult supports and, thus, that young people need to be given tools to help them assist victims through emotional support and image deletion in non-judgemental ways. As Henry et al. (2017) argue, to holistically respond to NCIID, there is an ‘urgent need’ among all age groups for community education campaigns and information resources to

meet the information and support needs of victims; encourage ‘witnesses’ or ‘bystanders’ to take action to support a victim and/or challenge the perpetrator; [and to] challenge the culture of victim-blaming that both excuses perpetrator behaviour and prevents victims from seeking assistance. (p. 1)

Given what young people revealed about dealing with instances of NCIID within their peer groups, we believe that educational approaches must be designed in collaboration with young people in order to challenge the cultures that contribute to blaming and shaming responses to image-sharing practices. A positive Canadian example of such collaborative work can be found at webwise.ca.^6^ Some of the young people interviewed for this research also mentioned the resource Healthy Relationships for Youth (HRY), a community-based peer education programme in their area that utilizes young people as facilitators. HRY is described as ‘a tool for schools to start preventing violence by addressing the underlying issues of sexism, racism, classism, ableism, transphobia, and homophobia’ and the ‘curriculum supports the goal of violence prevention by giving students the information and space to express their thoughts and emotions’ (Healthy Relationships for Youth Curriculum, 2019: 3). The programme’s curriculum includes a session titled ‘Power and Violence’ which encourages students to think critically about healthy and unhealthy relationships, and consent. During this session, facilitators discuss sex-positive ways to handle intimate image sharing situations and engage peers in positive discussions about how to navigate instances where the sharing is non-consensual. Anonymous help lines, such as Kids Help Phone in Canada, are also likely valuable resources as they provide young people advice for dealing with these issues in a confidential environment. Importantly, some helplines^7^ have also moved towards a model, aligned with sex-positive feminist approaches (Albury et al., 2016; Karaian, 2014), that educate young people about NCIID without implying that victims should be ashamed or should have known better if they consensually shared their intimate image.

In those rare cases among young people that are currently being reported to adult supporters, many of these supporters express that non-criminal alternatives should be made available that allow for expedient, individualized, victim-centred and restorative responses. The work of restorative/transformative justice scholars and anti-carceral feminist scholars provides a road map for understanding the efficacy of restorative, informal and/or victim-centred responses to sexual violence outside of the criminal law. For instance, as has been shown in regard to sexual violence more broadly, restorative and transformative justice practices could offer victims of NCIID response options that are better aligned with their particular needs and that, rather than being focused on punishing perpetrators, are focused on ‘elevating the voice of the victim or survivor’, addressing the harms caused and reintegrating ‘all parties back into the community’ in a healthy way (Kim, 2018: 226; Llewellyn, 2019). School-wide anti-discrimination and consent education could also address the student cultures that often cause and preserve the harm of non-consensual image sharing through victim blaming and shaming and could offer more opportunity to educate potential perpetrators and bystanders (Shariff and DeMartini, 2015). In addition, victims should be able to access non-judgemental mental health supports and supports for removing intimate images without being made to engage in a formal criminal justice process to do so. In response to the often-related issue of cyberbullying, Coburn et al. (2015) argue that ‘the costs of investigating, prosecuting, and sanctioning youth who engage in cyberbullying would be better placed in prevention and early intervention programs to reduce cyberbullying’ and in ‘mental health services for young people who might be at risk for depression, anxiety, self-harm, and suicide’ (p. 575). Likewise, a focus on criminal justice responses to teenagers’ acts of NCIID may move resources away from alternatives that our findings show might be more appealing to teenage victims and their supporters.

Shariff and DeMartini (2015) express concern that the creation of new criminal laws to respond to NCIID may provide a false sense that this issue has been adequately addressed. They argue that, contrary to this belief, cultural change and multipronged approaches are needed to truly prevent this act and support victims. Heeding this concern, activists, educators and policymakers should not treat this issue as solved and should continue to seek solutions on other fronts. Our findings provide evidence that young people may not see criminal justice responses as a useful tool and, in fact, may avoid seeking adult support more broadly due to fears of adult overreaction, victim blaming and shaming, and self/peer criminalization. The perspectives of our survey respondents and interviewees suggest the need to consider implementing more non-criminal support options in response to NCIID and to ensure that supports and education are provided in a non-judgemental manner.

## Notes

1. Non-consensual intimate image distribution (NCIID) refers to the act of distributing nude, partially nude or sexually explicit images of someone without their consent. This term does not encompass acts of image distribution committed by adults against minors. Thus, when referring to NCIID among young people, we are referring to acts committed between close-in-age young people that can legally engage in sexual activity with each other.

2. In Canada, for instance, the case of Canadian teenager Rehtaeh Parsons is regularly referred to as the catalyst for creating criminal responses to NCIID. Parsons died by suicide in the aftermath of having an intimate image of her (captured during an alleged sexual assault) non-consensually distributed and used as fodder for bullying and harassment.

3. *Criminal Code* s. 162.1: ‘Everyone who knowingly publishes, distributes, transmits, sells, makes available or advertises an intimate image of a person knowing that the person depicted in the image did not give their consent to that conduct, or being reckless as to whether or not that person gave their consent to that conduct, is guilty (a) of an indictable offence and liable to imprisonment for a term of not more than five years; or (b) of an offence punishable on summary conviction’ (R.S.C., 1985, c. C-46).

4. See https://needhelpnow.ca/app/en/resources_involving_safe_adult.

5. This finding echoes similar findings in terms of young people’s perspectives on responses to the sometimes related issue of cyberbullying (Choo, 2015).

6. See https://webwise.ca/cyber-101/sexting/

7. See, for example, Kids Help Phone (2019) at https://kidshelpphone.ca/get-info/what-sexting/ and Webbing with Wisdom (2016) at https://webwise.ca/cyber-101/sexting/.
